# Relative expression of receptors in uterine natural killer cells compared to peripheral blood natural killer cells

**DOI:** 10.3389/fimmu.2023.1166451

**Published:** 2023-03-24

**Authors:** Nurul Izza Ismail

**Affiliations:** School of Biological Sciences, Universiti Sains Malaysia, Gelugor, Penang, Malaysia

**Keywords:** uterine natural killer (uNK) cells, NK receptor, natural killer cell (NK cell), immune cell, peripheral natural killer (pNK) cell

## Abstract

One would expect maternal immune cells to attack the invading trophoblast as the placenta is semi-allogenic. However, they appear to cooperate with the trophoblast in disrupting the arterial wall which has been determined in several studies. uNK cells are a particular type of immune cell that appears to play a role in pregnancy. As in pregnancy, the key contributors to trophoblast invasion appear to be a unique combination of genes, which appear to regulate multiple components of the interactions between placental and maternal cells, called HLA class 1b genes. The HLA class 1b genes have few alleles, which makes them unlikely to be recognized as foreign by the maternal cells. The low polymorphic properties of these particular HLAs may aid trophoblasts in actively avoiding immune attacks. This review gives a complete description of the mechanisms of interaction between HLAs and maternal uNK cells in humans.

## Introduction

A major component of the mother’s leukocytes population is composed of uterine natural killer (uNK) cells, which are approximately 70% of the total decidual leukocyte population in the first trimester of pregnancy (1–3). uNK cells are lymphocytes that survey the body for abnormal cells. uNK cells are present in low numbers even before pregnancy, during the menstrual cycle’s proliferative and early secretory phase. The number of uNK cells in the endometrium increases through the late secretory phase of the menstrual cycle and continues to rise in early pregnancy. The uNK cells accumulate in the decidua until week 20 of gestation, with the highest numbers present in early pregnancy.

uNK cells play a unique role in decidualization and trophoblast implantation, and uNK cells have been suggested to be remarkably different from other subsets of CD56^bright^ NK cells in peripheral blood (4–10). The origin of uNK cells remains unclear, but uNK cells (CD56^bright^CD16^dim^) have been detected in both non-pregnant and pregnant uterine tissue (9, 11). uNK cells have been found to secrete different types of cytokines compared to peripheral NK (pNK) cells and are poorly cytotoxin in normal pregnancies (12). uNK cells do appear to produce low proportion of cytotoxic proteins, but this is not thought sufficient to kill the invading trophoblasts (13). Similar to cytotoxin secretion, secretion of cytokine is also influenced by the activating and inhibitory receptors on the surface of uNK cells.

## Regulation and mechanism of NK cell function

NK cell function is determined by a balance of activation and inhibition signalling induced by trans-membrane receptors (14, 15). Integration of these numerous inputs results in cytotoxin and/or cytokine secretion. This process involves the engagement of ligands with the receptors, as well as the action of pro-inflammatory cytokines such as IL-1, IL-2, IL-12, IL-15, IL-18, IL-21 and IFNα,β (8, 16, 17) released by antigen-presenting cells (APCs) (18). NK cells do not have a dominant activation receptor, so several receptors or coactivations are required to secrete cytotoxins. If activation signalling dominates, NK cells become activated. Following activation, an NK cell can secrete cytotoxins and various types of cytokines. Cytotoxin secretion is suppressed by the recognition of ligands that the NK cell receptors detect on ‘target’ cells (19). Upon contact with other cells recognised as undesirable, activated NK cells are able to mediate cell killing *via* two mechanisms, exocytosis of perforin/granzyme granules and signalling *via* TNF receptors (8, 19).

**Figure 1:**
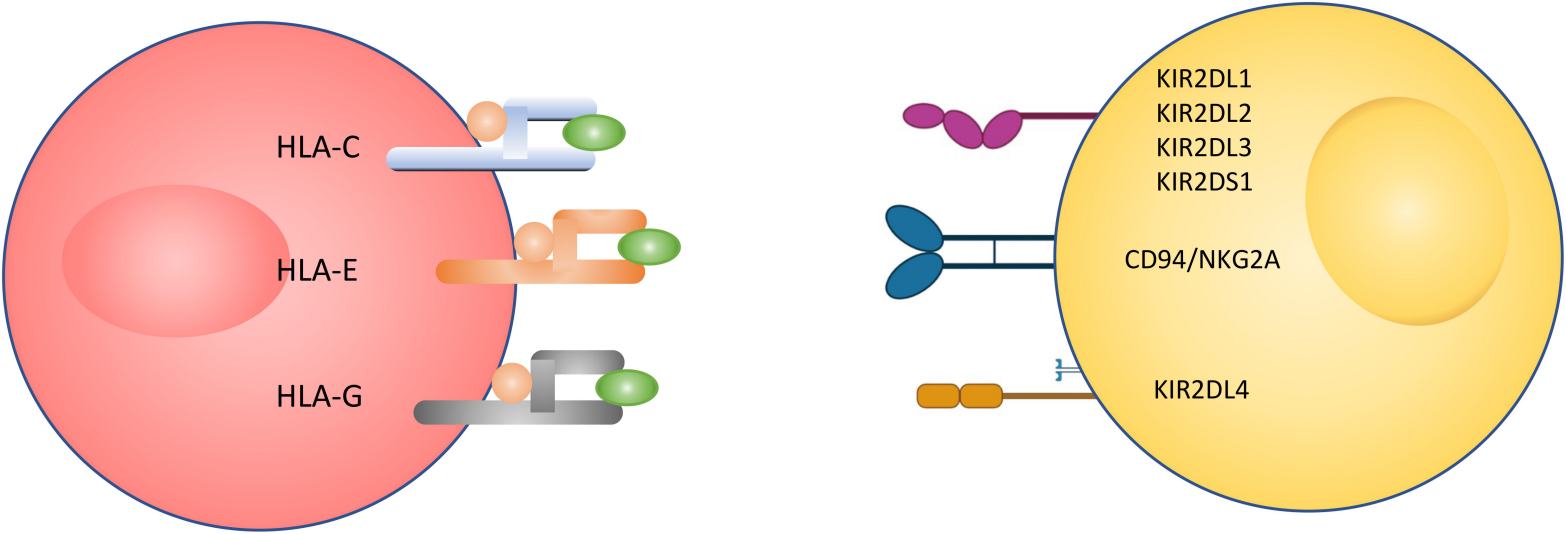
Illustration of NK cell interaction with a target cell. The interaction between the two cells happens through receptors expressed on the NK cell surface and ligand expressed on the target cell surface.

The interaction between NK cells and target cells requires docking between NK cell receptors and target cell ligands (**Figure 1**). There are two hypotheses regarding how NK cells interact with pathogens or foreign cells. The ‘missing-self’ hypothesis is one of the simple recognition strategies for NK cells. This hypothesis suggests that NK cells attack target cells that lack ‘self-molecules’, which usually exist in a healthy or normal cell (20). The action of NK cells can be described as ‘activation upon recognition of the unexpected and inactivation upon recognition of the expected’. Another recognition strategy is called the ‘induced self’ hypothesis, where NK cell activation happens because of the expression of ligands for NK cell-activating receptors. The activation of NK cells in this situation is induced under cellular stress conditions, like viral infection (21). Both recognition methods may work simultaneously for NK cells to maximally discriminate between normal cells and infected target cells (22).

NK cells can be divided into two distinct populations: CD56^dim^ and CD56^bright^, which differ in their distribution of homing properties. CD56^dim^ makes up 90% of human peripheral blood, while CD56^bright^ represents the main lymphocyte population in human decidua (10). Normally CD56^dim^ cells express high levels of CD16 (23), the strongest inducer of cytotoxicity by IL-2 activated NK cells (17). While CD56^bright^ cells express no, or low levels, of CD16. So, CD56^dim^ and CD56^bright^ cells secrete different types or levels of cytotoxins and cytokines (6, 16). As there is a distinction between the NK cells based on both CD56and CD16, the population of NK cells is also recognized as being split into CD56^dim^CD16+ and CD56^bright^CD16-cohorts. Another difference between cells in the two populations of CD56is perforin expression. It is expressed in high levels in CD56^dim^, while CD56^bright^ cells express 10-fold lower perforin (16, 23).

## Natural killer cell receptors

**Figure 2:**
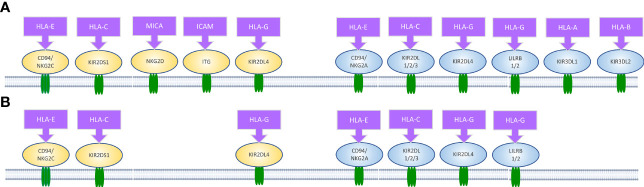
Receptors of interest in human natural killer cell. Activating (highlighted in yellow) and inhibitory (highlighted in blue) receptors expressed on **(A)** pNK and **(B)** uNK surfaces, and ligands that bind to them (24–28).

A large number of NK cell receptors have been studied and the number is still growing (**Figure 2**) (6, 29–32). There are three major families of natural killer receptors (NKRs). The first family is the killer cell Ig-like receptor (KIR). This group of receptors recognizes human leukocyte antigen (HLA) -A, -B and -C on target cells. The expression of KIR isoforms on NK cells is regulated by the methylation of KIR gene loci (21). A second group is C-type lectins, such as CD94 and NKG2, which recognize HLA-E and MICA (ligands on tumour, infected and/or stressed cells) on target cells. A third group is the natural cytotoxicity receptors (NCRs) which comprise activation receptors to signal cells’ lysis. Among them are NKp44, Nkp46, NKp80 and NKp30 (21), which interact with viral hemagglutinin, nuclear factor HLA-B-associated transcript 3, heparin sulphate proteoglycans, C-type lectin and B7-H6 (21). There is a small family of receptors in humans, called leukocyte immunglobulin-like transcript (ILT) receptors, which are inhibitory receptors that bind to the HLA class I ligand.

The KIR receptor group is of particular interest in this study because of its capacity to bind with HLA-G and HLA-C, which have both been found to play important roles in trophoblast invasion. Commonly, inhibitory KIRs contain ITIM (immunoreceptor tyrosine-based inhibition motifs) regions in their cytoplasmic tail, and activation KIRs contain ITAM (immunoreceptor tyrosine-based activation motif) regions in their cytoplasmic tail. ITIM is defined by a consensus of amino acid sequence (I/L/V/S) xYxx (L/V), where the x represents any amino acid. Once an inhibition receptor engages with a target cell ligand, the tyrosine residue on the ITIM is phosphorylated and activates inhibition signalling downstream. In contrast, the ITAM consensus sequence is defined by (D/E)xxYxx(L/I)x(6-12)Yxx(L/I), where x(6-12) represents 6-12 possible amino acids (some say x(6-8)) (30, 31, 33). Upon engagement with the ligand on the target cell, the tyrosine residue is phosphorylated by Src family protein tyrosine kinase (SFK). This initiates the activation signalling pathway of an NK cell (30, 34).

## NK cells in decidua

### Trophoblasts and the major histocompatibility complex: A ligand and receptor interaction

MHCs or HLAs are normally highly variable between individuals, as they are encoded by numerous highly polymorphic genes, meaning that it is extremely unlikely that two people will possess cells with the same set of HLA molecules. There is strict regulation of HLA genes, and production of their proteins, by invading trophoblasts (35). While there are multiple mechanisms that may contribute to immune tolerance in pregnancy, a particular class of non-classical HLA-E and -G, and classical HLA-C appear to play a key role in maternal immune response. Another important mechanism by which the fetus avoids the maternal immunologic response is the failure of trophoblasts to express classical HLA class Ia, comprising HLA-A and -B. Without expression of HLA-A and -B molecules, trophoblasts are unlikely to be killed by NKs through secretion of cytotoxic. In contrast to classical HLA (HLA-A, -B and -C), there is limited variability between individuals in HLA-E and -G (35, 36). HLA-E and -G have low numbers of alleles that differ at the protein level, HLA-E has 2 alleles and HLA-G has five alleles that do not alter the amino acid sequence (35). It is suggested that because of the limited variability in HLA-E and -G that uNK cells are “poor killers of the usual NK cell targets” (35). HLA-E, HLA-C and more so HLA-G, are thought to be highly expressed in placental cells to 1) provide recognition for uNK cells that is not highly variable between individuals, and 2) to inhibit the toxicity of uNKs. However, the true nature of the interaction between invading placental cells and uNK cells is not well understood. In this section we will address HLA-G, -C and -E and their potential roles in pregnancy.

Most of the natural killer cell receptors that have potential ligands expressed by extravillous trophoblasts (EVTs) have been described. HLA-G binds to members of the LILR family, including LILRB1, LILRB2 and KIR2DL4. HLA-E binds to NKG2C and its inhibitory counterpart NKG2A. Classical HLA-C allotypes that are polymorphic bind to members of the KIR2DL/S family (24). While NKG2C and KIR2DS1 are activating, LILRB1/2, KIR2DL1/2/3 and NKG2A are inhibitory. In contrast, KIR2DL4 can be either activating or inhibitory depending on the amino acid residues in the domain. During the first 8-10 weeks of gestation, KIR2DL1/2/3 and KIR2DS1 expression on uNK cells is very high compared to pNK cells from the same women at the same time, and NKG2A is expressed on almost all uNK and LILRB1 is expressed on 30-40% of uNK cells. This suggests a majority of uNK cells may bind to HLA-C expressed by EVTs (24, 37, 38).

### Relative expression of receptors in uNK cells compared to pNKs

HLA-G is the MHC complex that is most abundant during term pregnancy. HLA-G stimulates uNK cells’ secretion of cytokines and further induces immune tolerance, controlling EVT invasion, and contributing to vascular remodelling of the spiral arteries (39). HLA-G is a homodimer expressed almost exclusively by EVTs (40). HLA-G has eight exons and a specific feature of HLA-G is the seven alternative splicings that permit the formation of various isoforms of HLA-G; four membrane-bound and three soluble proteins. The highly expressed HLA-G isoforms in trophoblasts during early pregnancy are membrane-bound HLA-G1 and soluble HLA-G5. While mRNAs for HLA-G4 and G7 are not abundant in placentas (35), HLA-G is readily detected in the EVTs that invade the maternal endometrium (14, 41). Soluble HLA-G has been detected in the peripheral blood and other biological fluids of pregnant women (42). Additional studies reported that soluble HLA-G was detected in the culture medium from human IVF-derived embryos. The presence of HLA was significantly associated with subsequent pregnancy following transfer to recipients (43–46).

In the uterus, HLA-G appears to have important interactions with uNK cell surface receptors, in particular the killer cell immunoglobulin-like receptor (KIR2DL4) and plays an important role in placental development. Rajagopalan & Long (47) demonstrated that KIR2DL4 interacts with cells expressing HLA-G but not with cells expressing HLA class I molecules. Unlike other KIR members, KIR2DL4 is expressed by all NK cells. KIR2DL4 is a unique KIR member since it has both activation and inhibition features (29, 48). KIR2DL4 has an ITIM region and also a positively charged arginine amino acid residue in the transmembrane region that is a feature of activation receptors. Therefore, many describe KIR2DL4 as an activating receptor with inhibition potential (22, 32, 47–49). KIR2DL4 and HLA-G ligation and treatment of uNK cells produce a proinflammatory cytokine, interferon- (IFN).

Another important MHC molecule in pregnancy that is expressed by EVTs is the classical class Ia HLA-C molecule (7). Although HLA-C is moderately polymorphic, both paternal and maternal alleles of HLA-C are expressed by trophoblasts, which could reserve the semi-allogenic characteristics of HLA-C (7). In addition, the allelic disparity at the HLA-C locus is improbable to cause failed pregnancy (35). Hiby et al. (50) suggested an imbalance in stimulation of uNK immunoglobulin-like receptors (KIR) by HLA-C in pre-eclampsia in human pregnancy (50). KIR specificity for HLA-C is expressed in high density on uNK cells compared to pNK cells in pregnant women, indicating the high density of HLA-C expressed by uNK cells.

HLA-C is the only classical HLA known to be expressed in trophoblasts. Because both KIR and HLA-C are polymorphic, maternal KIR and fetal HLA-C genetic combination can vary between pregnancies (50). KIR exists in two groups, A and B, which are specified as inhibiting and activating uNK, respectively. The KIR haplotype A is a simpler and mainly inhibitory KIR, while the B KIR haplotype is complicated and mainly activating. Women with homozygous AA have a higher risk of pre-eclampsia than those who are BB and heterozygous AB (50). HLA-C allotypes exist in two groups; HLA-C1 and HLA-C2, according to the particular amino acid residue at position 80 (51). HLA-C1 has asparagine (Asn) and acts as a ligand for inhibitory KIR2DL2 and KIR2DL3. HLA-C2 has a lysine (Lys) at residue 80 and is a ligand for the inhibitory KIR2DL1 receptor and the activating KIR2DS1 receptor. HLA-C2 is a stronger ligand than HLA-C1 upon interaction with the receptors (50).

A combination of fetal HLA-C2 and a maternal AA KIR genotype will most probably lead to pre-eclampsia and poor placentation (29, 50, 52). As an example, HLA-C2 and KIR2DL1 binding is expected to cause pre-eclampsia. Overly inhibited uNK cells may cause EVTs to prematurely stop the remodelling of spiral arteries and this inadequate remodelling thereby increases the risk of pre-eclampsia (52). Therefore, Hiby et al. (50) suggested that high inhibition signalling in uNK cells favours pre-eclampsia. However, the strong uNK cells inhibition could be balanced by the interaction of HLA-C2 with activation KIR AB/BB genotypes receptors. Parham (52) suggested that activating KIR lowers the likelihood of pre-eclampsia and the absence of activating KIR favours pre-eclampsia.

A recent study indicated that human birth weight is regulated by the interaction between maternal KIR and paternal HLA-C2 (50). This suggests that the frequent inhibitory signals in maternal KIRs produce small babies but frequent activating KIR signals result in big babies, compared to normal growth. Thus, the balance between activation and inhibition contributes to successful pregnancy by maintaining the fetus birth weight between the two extremes (53). Investigations have shown that KIR2DS1 is the gene with the most significant effect on human birth weight. Although KIR2DS5 has also been shown to increase birth weight, the impact is not significant (50).

There is a high expression of inhibitory receptor CD94/NKG2A on uNK cells, which suggests the HLA-E ligand exists in maternal decidua (7). The interaction between CD94/NKG2A and HLA-E is believed to prevent the lysis of both maternal and paternal tissue cells in the vicinity of uNK cells. HLA-G complexes with HLA-E and binds CD94/NKG2C, giving enough affinity to trigger an NK cell response. This means that HLA-G needs HLA-E to influence the maternal immune response. This mechanism will still occur even if the fetus is homozygous for the HLA-G null allele because the leader peptide will continue to be translated (51, 54). HLA-E could be the major inhibitory regulator for uNK cells, rather than HLA-G (1). Knowing that HLA-E is the only ligand for the CD94/NKG2A heterodimer, this suggestion is supported by the large amount of CD94/NKG2A found in the maternal decidua (35, 55).

Inhibitory receptors regulate the activities of various activating receptors and have diverse signalling pathways downstream. NKs are inhibited by inhibition receptors that recognize MHC class I. Inhibitory receptor engagement reduces tyrosine phosphorylation. For example, the CD94/NKG2A inhibition receptor complex recognizes HLA-E, which results in phosphorylation of HLA-E and CD94/NKG2A complex by SFKs. This further leads to the recruitment of phosphatase SHP to the NKG2A complex (at the ITIM region). A common feature of inhibition receptors involved in these protective functions is the presence of ITIM in the cytoplasmic tail. The functional consequence of SHP recruitment by ITIM-containing receptors in NK cells is well established. Vav is a direct target of SHP when SHP is recruited to the ITIM region. Signals from inhibitory receptors dephosphorylate Vav through SHP. However, coaggregation between activating and inhibitory receptors is required for inhibition to occur.

The known differences in receptor-ligand interactions between pNKs and uNK cells are illustrated in **Figure 2**. As described earlier, the important ligands expressed on trophoblasts are HLA-C, -E and -G. These are the ligands that therefore are assumed determine the response of uNKs to trophoblasts. As reported in the literature, we hypothesised that the level of cytokine secretion is increased in uNK cells compared to pNK cells.

Papuchova et al. (56) analysis of the HLA-C, -E, and -G differential expression shows that HLA-C and -E expression during term pregnancy (>37 weeks) was significantly lower compared to the first trimester (6-12 weeks). Meanwhile, the first trimester EVT has the highest levels of HLA-C and term pregnancy EVT has the highest levels of HLA-G. These differences suggest that EVT has a distinct ability for antigen presentation and interaction with uNK at different stages of pregnancy (57).

### uNK cytokine and chemokine secretion

Generally, IFNγ and TNFα are the cytokines secreted in the highest levels by NK cells (25, 30). NK cells also secrete GM-CSF (23, 58). For particular NK receptors, there is evidence that KIR2DL4 receptor activation by HLA-G induces the production of IFNγ and TNFα (48, 58, 59). The production of IFNγ by KIR2DL4 is blocked by inhibition of MAPK pathway. Experimental work by Sharkey (2008) showed that KIR activation includes KIR2DS1 activation by HLA-C and results in increased secretion of IFNγ (40). HLA-E and HLA-G also stimulate the secretion of IFNγ, TNFα and GM-CSF in large granular lymphocytes (LGL) (56).

## Discussion

Immune cells have been found aggregated around spiral arteries in early pregnancy, near the invading trophoblast (60–62). Smith et al. (62) and Rana et al. (63) suggested that spiral artery remodelling is initiated by uNK cells. A reduced number of uNK cells have been detected in pregnant mothers with pre-eclampsia and intrauterine growth restriction (IUGR), which is believed to cause poor spiral artery remodelling (2). The key contributors to trophoblast invasion appear to be a unique combination of HLA genes. The low polymorphic properties of these particular HLAs may aid trophoblasts in actively avoiding immune attacks (64). We concluded that the uNK lack of the ligand-receptor pairs that are reported in pNK which are ICAM, MICA, HLA-A and HLA-B ligands and their respective receptors ITG, NKG2D, KIR2DL1 and KIR2DL3.

## Author contributions

The author confirms being the sole contributor of this work and has approved it for publication.
